# Polyethylenimine-based iron oxide nanoparticles enhance cisplatin toxicity in ovarian cancer cells in the presence of a static magnetic field

**DOI:** 10.3389/fonc.2023.1217800

**Published:** 2023-09-12

**Authors:** Faranak Ashoori, Behnam Hajipour-Verdom, Mohammad Satari, Parviz Abdolmaleki

**Affiliations:** ^1^ Department of Biophysics, Faculty of Biological Sciences, Tarbiat Modares University, Tehran, Iran; ^2^ Department of Biology, Faculty of Sciences, Malayer University, Malayer, Iran

**Keywords:** magnetic iron oxide nanoparticles, cisplatin, polyethylenimine, cytotoxicity, cell responses

## Abstract

**Background:**

Drug resistance in cancer cells is a major concern in chemotherapy. Cisplatin (CIS) is one of the most effective chemotherapeutics for ovarian cancer. Here, we investigated an experimental approach to increase CIS cytotoxicity and overcome cell resistance using nanoparticle-based combination treatments.

**Methods:**

Polyethylenimine (PEI)-based magnetic iron oxide nanocomplexes were used for drug delivery in genetically matched CIS-resistant (A2780/CP) and -sensitive (A2780) ovarian cancer cells in the presence of a 20 mT static magnetic field. Magnetic nanoparticles (MNPs) were synthesized and bonded to PEI cationic polymers to form binary complexes (PM). The binding of CIS to the PM binary complexes resulted in the formation of ternary complexes PM/C (PEI–MNP/CIS) and PMC (PEI–MNP–CIS).

**Results:**

CIS cytotoxicity increased at different concentrations of CIS and PEI in all binary and ternary delivery systems over time. Additionally, CIS induced cell cycle arrest in the S and G2/M phases and reactive oxygen species production in both cell lines. Ternary complexes were more effective than binary complexes at promoting apoptosis in the treated cells.

**Conclusion:**

PEI-based magnetic nanocomplexes can be considered novel carriers for increasing CIS cytotoxicity and likely overcoming drug resistance of ovarian cancer cells.

## Introduction

Disruption of the cell cycle progression impacts cell homeostasis and physiological functions, potentially leading to cancer. Ovarian cancer is the fourth most commonly diagnosed cancer among females globally and ranks first among cancers that threatens the female reproductive system. Several risk factors for ovarian cancer, such as lifetime ovulatory cycles, have been identified in previous studies (1, 2). Unfortunately, ovarian cancer is often diagnosed at an advanced stage when the tumor has already spread beyond the ovary, making it the leading cause of death among gynecologic cancers (3).

Cisplatin (CIS) platinating agents are commonly used in chemotherapy against various types of breast, lung, and ovarian cancers (4–6). This compound inhibits DNA synthesis by binding to the guanine base and forming intra-strand DNA adducts (4). Drug resistance is the most critical problem in the CIS treatment of ovarian tumor cells that occurs in the early phase of chemotherapy (7, 8). Various mechanisms of drug resistance have been proposed, including genetic polymorphisms in copper transporter proteins (9–11). Another mechanism that leads to CIS drug resistance is the multidrug resistance (MDR) protein, which pumps the drug extracellularly with energy consumption (12–16). Generally, cellular adaptations can accomplish CIS resistance, including uptake decrement, inactivation by glutathione, metallothionein, and other antioxidants, and increased DNA repair gene expression (15, 17–19). There are ongoing research efforts to overcome drug resistance, which are necessary for efficient treatments. However, currently, there is no practical approach to overcome drug resistance in cancer therapy (20–25).

Using Hsp90 inhibitors, Zhang et al. reversed CIS resistance by modifying the expression of multiple drug resistance-related genes (26). In another study, Ai et al. showed that inhibition of HIF-1α induces reactive oxygen species (ROS) overproduction in CIS-resistant cells and resensitizes CIS-resistant ovarian cancer cells (27). Moreover, Hu and Zhang found that targeted nanocarriers can deliver drugs, oligonucleotides, peptides, and DNA to tumor cells by enhancing permeation and retention (28). Nanoparticle-based combination approaches used in clinical assessment to overcome drug resistance include control over drug loading, temporary sequencing of drug release, and co-encapsulation of drugs with various physicochemical properties (28, 29).

Studies have demonstrated that curcumin-loaded nanoparticles induce apoptotic cell death by regulating MDR functions and ROS levels in CIS-resistant human ovarian cancer cells (30). Polyethylenimine (PEI) polymer has been used to transfer genes, proteins, and anticancer drugs (31–35). Yang et al. showed that chemo-radioresistance of glioblastoma decreased using microRNA (miR)145 with cationic polyurethane-short branch PEI (PU-PEI). PU-PEI-miR145 delivery effectively suppressed the expression of drug-resistance and antiapoptotic genes and can consequently lead to a novel therapeutic approach for malignant brain tumors (36).

Magnetic fields (MFs) affect biological systems and may increase ROS production, leading to oxidative stress in DNA, proteins, and lipids and cause genetic mutations and cell death (37–39). Anticancer agents and MFs can impact cell proliferation, and their combination may provide a novel approach in cancer therapy to enhance the anticancer effect of chemotherapeutics (22, 37, 40). This study investigated the potential of magnetic iron oxide nanoparticles (MNPs) with a PEI cationic polymer to increase CIS cytotoxicity and overcome drug resistance. These drug-loaded PEI-based MNPs were delivered using a homogenous static magnetic field (SMF) into sensitive and resistant ovarian cancer cell lines. The PEI-based magnetic nanocomplexes can boost drug delivery and cytotoxicity and may decrease cell drug resistance.

## Materials and methods

### Chemical reagents

Roswell Park Memorial Institute (RPMI)-1640 medium and fetal bovine serum (FBS) were purchased from Gibco. Penicillin–streptomycin and trypsin–EDTA were purchased from Bioidea. Cisplatin was purchased from Oncotec Pharma Production GmbH (Germany). Branched polyethylenimine (25 kDa) was purchased from Sigma-Aldrich. 2′,7′-Dichlorofluorescein diacetate (DCFDA, ab113851) was purchased from Abcam. Propidium iodide (PI), iron (II) chloride tetrahydrate (4H_2_O.FeCl_2_), iron (III) chloride hexahydrate (6H_2_O.FeCl_3_), and sodium hydroxide (NaOH) were purchased from Merck. Annexin V apoptosis detection kit was purchased from eBioscience (USA).

### Synthesis and characterization of magnetic nanoparticles

Fe_3_O_4_ MNPs were synthesized using the co-precipitation approach. The properties of nanoparticles and size-dependent parameters, such as reaction temperature, suspension pH, and initial molar concentration, have been investigated (41, 42). In this approach, nanoparticles are synthesized from 4H_2_O.FeCl_2_, 6H_2_O.FeCl_3_, and NaOH with high purity and distilled water. The 4H_2_O.FeCl_2_ and 6H_2_O.FeCl_3_ solutions were mixed in the respective stoichiometry (i.e., 1:2 ratio of Fe (II):Fe (III)) in presence of OH¯ deposited through the following reaction in Equation (1).


(1) $${\text{Fe}}^{2+}+{\text{ Fe}}_{\;}^{3+}+8{\text{OH}}^{-}\to \text{Fe}{\left(\text{OH}\right)}_{2}+2\text{Fe}{\left(\text{OH}\right)}_{3}\to {\text{Fe}}_{3\;}{\text{O}}_{4}+4{\text{H}}_{2}\text{O }$$

The ultrasonic transducer and nitrogen gas initially deoxygenated distilled water. Iron salts II and III were then added. The ultrasonic apparatus also deoxygenated the NaOH solution. Twelve microliters of saline solution were added to 120 mL of NaOH solution under nitrogen atmosphere, and the solution was homogenized for 10 h at 10,000 rpm for 30 min. Subsequently, the particles were washed with distilled water and once with acetone, and dried under vacuum conditions (43, 44).

The binary complexes PEI–CIS (PC) and PEI–MNPs (PM) were synthesized at 37°C for 1 h. To form ternary complexes [PEI–MNP/CIS (PM/C)], CIS bound to PM binary complexes. Moreover, PEI, MNP, and CIS bound together simultaneously, and PEI–MNP–CIS (PMC) ternary complexes were synthesized at 37°C for 1 h.

The accuracy of MNPs and the formation of binary and ternary complexes were confirmed by Fourier transform infrared spectroscopy (FTIR) and dynamic light scattering analysis. FTIR spectra were obtained for a dried sample of MNP, PM binary, and PMC ternary complexes using an FTIR spectrometer (NICOLET IR100; Thermo Scientific) in a wave-number range of 4000–400 cm^-1^ with a resolution of 4 cm^-1^. In brief, the dried sample was placed on a silicon substrate transparent to infrared, and the spectra were measured using the transmittance method. The spectra of synthesized products were then plotted using the Essential FTIR software. The size and surface charge distribution of the PEI, MNP-PM, and PMC ternary complexes in solutions and suspensions were determined using dynamic light scattering (DLS Zetasizer, a nano-ZS model; UK). Briefly, a 1 mg/mL solution of nanoparticles was prepared in deionized water and placed in an ultrasonic bath for 30 min. The sample was filtered using a 0.25 μm filter to remove larger and accumulated particles. Then, the particle size distribution and surface charge were analyzed using the Zetasizer software (version 7.11).

### Cell culture

The CIS-resistant human ovarian carcinoma A2780/CP and sensitive A2780 cells were obtained from the National Cell Bank of Iran (NCBI). A2780/CP is a sub-line of A2780 that gained CIS resistance *in vitro* (27). The cells were allowed to grow in RPMI-1640 in neutral PH (7.2–7.4) supplemented with 10% (v/v) heat-inactivated (50°C, 30 min) FBS and 2 mM glutamine, 100 units/mL of penicillin and 100 mg/mL of streptomycin at 37°C and 5% CO_2_ in a humidified incubator. The cells were trypsinized (0.025% trypsin and 0.02% EDTA) after reaching 70–80% confluency. Prior to treatments, cells were allowed to reattach overnight.

### Magnetic field exposure

We used permanent cobalt magnets to apply a 20 millitesla (mT) homogenous SMF. The magnitude of this magnetic field was calculated using a Teslameter (13610.93; PHYWE, Gottingen, Germany) with a Hall effect probe regarding its cross-sectional area and thickness. During this study, we exposed the cells to 20 mT SMF by placing magnets under the bottom cell culture plates.

### Cell treatments

The A2780/CP and A2780 cells were treated with CIS and PEI at concentrations of 5, 10, 25, 50, and 100 μg/mL; the PC binary complex comprised various concentrations of PEI (1, 2.5, 5, and 10 μg/mL) and CIS (2.5, 5, and 7.5 μg/mL) and the PM binary complex, of PEI (2.5, 5, and 10 μg/mL) and MNPs (1 μg/mL) in the presence and absence of 20 mT SMF for 24 and 48 h. Moreover, the cells were treated at a mass ratio of PEI/CIS (0.4, 0.5, 1, 2, and 4 w/w) for 24 and 48 h. In addition, the cells were treated with PM/C and PMC ternary complexes at concentrations of 1 μg/mL PEI, 1 μg/mL MNPs, and 2.5 μg/mL CIS in the presence and absence of SMF for 48 h.

### Cell viability assay

The viability of A2780/CP and A2780 cells was measured using a tetrazolium-based colorimetric (MTT) assay. Briefly, cells (10^4^ cells/well) were seeded in a 96-well culture plate (SPL Life Sciences Co., Ltd. Korea) and incubated in a total volume of 100 μL supplemented with RPMI at 37°C and 5% CO_2_ in a humidified incubator. The cells were allowed to attach overnight. Following the treatments, 100 μL FBS-free RPMI with 0.5 mg/mL MTT was added to each well and kept at 37°C for 4 h in the dark. Then, formazan was dissolved in 100 μL/well DMSO. The relative number of living cells in each group was measured at 570 nm using a microplate reader (uQuant MQX200; BioTek, USA). Cell viability results are shown as a percentage compared to the control cells. The half-maximal inhibitory concentration (IC_50_) was used to evaluate the sensitivity of the selected cell types.

### Quantitation of intracellular ROS accumulation

Intracellular ROS levels under normal and stress conditions were detected using a 2′,7′–DCFDA assay kit. The A2780/CP and A2780 cells were treated with 1 µg/mL PEI and 2.5 µg/mL CIS, as well as with PM/C and PMC complexes (at concentrations of 1 µg/mL PEI, 1 µg/mL MNP, and 2.5 µg/mL of CIS), compared to untreated cells, in the presence and absence of SMF for 48 h, which were prepared in RPMI medium supplemented with 10% FBS in 6-well cell culture plates (SPL Life Sciences Co., Ltd. Korea). After treatment, the cells were prepared immediately, according to the manufacturer’s instructions. Briefly, the cells were washed with PBS. The samples were then suspended in a conical test tube with 20 µM DCFDA in 1X buffer and incubated at 37°C in the dark for 30–45 min. ROS production was monitored immediately using a FACSCalibur Becton-Dickinson flow cytometer (Franklin Lakes, NJ). DCFDA flow cytometric data were analyzed using FlowJo software (version 7.6.1) (22, 27, 45).

### Cell cycle analysis

The A2780/CP and A2780 cells were treated with 1 µg/mL of PEI and 2.5 µg/mL of CIS, and PM/C and PMC complexes (at concentrations of 1 µg/mL PEI, 1 µg/mL MNP, and 2.5 µg/mL CIS) in the presence and absence of SMF for 48 h, which were prepared in RPMI medium supplemented with 10% FBS in 6-well cell culture plates and incubated at 37°C and 5% CO_2_ in a humidified incubator. The cells were trypsinized and collected using centrifugation at 400 ×*g* for 5 min. Then, the cells were resuspended in 0.5 mL PBS, fixed by adding 4.5 mL of 70% (v/v) cold ethanol and centrifuged at 400 × *g* for 5 min. The cells were washed in 5 mL PBS, centrifuged at 400 × *g* for 5 min, incubated at room temperature for 5 min, and recentrifuged at 400 × *g* for 5 min. Then, the supernatant was removed and resuspended in 1 mL of DNA staining solution. The prepared cells were incubated for at least 30 min at room temperature in the dark. The obtained cell suspension was analyzed using a FACSCalibur Becton-Dickinson flow cytometer. Data were collected from at least 104 cells. The cytometric flow data were analyzed using FlowJo software (46, 47).

### Detection of cell apoptosis

Apoptosis was detected using annexin V-FITC/PI staining. The A2780/CP and A2780 cells were treated with 1 µg/mL PEI and 2.5 µg/mL CIS, and with PM/C and PMC complexes (at concentrations of 1 µg/mL PEI, 1 µg/mL MNPs, and 2.5 µg/mL CIS) in the presence and absence of SMF for 48 h, and were prepared in RPMI medium supplemented with 10% FBS in 6-well cell culture plates and incubated at 37°C and 5% CO_2_ in a humidified incubator. The cells were then collected and labeled with annexin V/PI in 1X binding buffer for 15 min. Apoptotic and necrotic cells were evaluated using a FACSCalibur Becton-Dickinson flow cytometer. The flow cytometric data were analyzed using FlowJo software. The total number of apoptotic and necrotic cells was defined as the sum of the Annexin V+/PI− and Annexin V+/PI+ populations; the cell populations in the four quadrants of the dot plot were analyzed as follows: the Q1 quadrant represented necrosis; Q2, late apoptosis; Q3, early apoptosis; and Q4 viable cells (47, 48).

### Statistical analysis

GraphPad Prism 5.0 (GraphPad Software Inc., San Diego, USA) was used for data graphing and estimating the values of IC_50_ for cytotoxicity. All experiments were performed in three independent repetitions. Data are shown as the mean ± standard deviation (SD) and analyzed using factorial analysis of variance (ANOVA) followed by Tukey’s *post hoc* tests. Differences were assessed to be significant for p-values >0.05.

## Results

### Formation of magnetic nanoparticles

**Figure 1:**
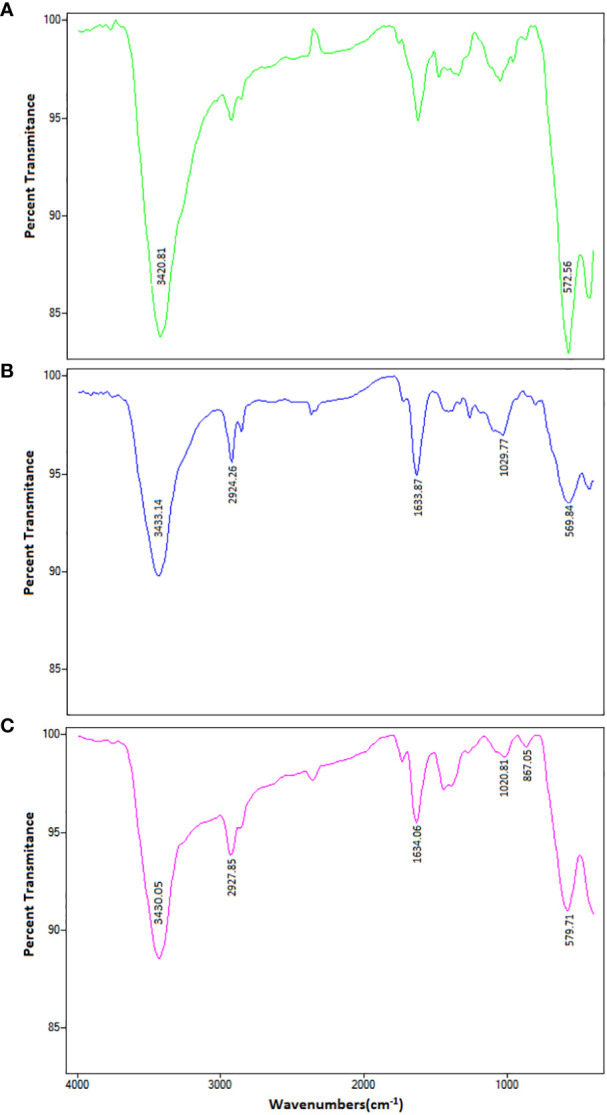
The FTIR spectrum of **(A)** MNPs, **(B)** PEI-MNPs (PM), and **(C)** PEI-MNP-CIS (PMC) in the range of 400-4000 cm^-1^ wavelengths. The FTIR spectrum of MNPs is visible on two peaks. The first absorption pic is 3420 cm^-1^ wavelength, related to the hydration bond formed with hydroxyl groups. The second absorption pic is 572 cm^-1^, due to Fe-O’s vibrational band in the nanoparticles. These peaks confirm the accuracy of nanoparticle synthesis. In the PM binary complex, in addition to MNPs-dependent peaks (3433 and 569 cm^-1^), the wavelengths 2924, 1633, and 1029 cm^-1^ indicate C-H, C-C, and C-N bonds of PEI polymer, respectively. These results confirmed the accuracy of PM binary complex formation. In the PMC ternary complex spectrum, the peaks of MNP and PM are in the range of 867 cm^-1^, which indicates the N-H bond of the cisplatin drugs.

As shown in **Figure 1**, the FTIR spectrum of MNPs is indicated by two peaks. The first absorption is at 3420 cm^-1^, which is related to the hydration bond formed with hydroxyl groups, and the second absorption is at 572 cm^-1^, which is due to the vibrational band of Fe-O in MNPs. These peaks confirm the accuracy of the MNP synthesis. In the PM binary complex infrared (IR) spectrum, in addition to the MNP peaks, the 2924, 1633, and 1029 cm^-1^ wavelengths indicate the C–H, C–C, and C–N bonds of the PEI polymer, respectively, confirming the accuracy of the PM binary complex formation. The FTIR spectrum of the PMC ternary complexes peaks at 867 cm^-1^, and the peaks of the MNPs and PM show that the N–H bond of CIS in conjugation with other molecules was<500 cm^-1^, which was not visible in our spectrum (44). Furthermore, the DLS results, presented in **Table 1**, revealed sizes corresponding to MNPs of ~ 69 ± 5 nm, PM binary complex of ~ 88 ± 15 nm, and PMC ternary complex of ~ 151 ± 21 nm, which appear suitable for delivery agents. Furthermore, the zeta potential results indicate that the MNP surface charge was approximately -25 eV, in which the negative surface charges create an interconnection through electrostatic interaction with the positive charges of PEI.

**Table 1:** Zeta potential and size related to Fe_3_O_4_ magnetite nanoparticles (MNPs), PEI-MNPs (PM) binary complex including MNPs and polyethylenimine (PEI) and three-component magnetic nanocomplexes (PMC) comprising PEI, MNP, and cisplatin.

	PEI	MNPs	PM	PMC
**Zeta potential (eV)**	34 ± 3	-25 ± 4	19 ± 2	16 ± 6
**Size (nm)**	91 ± 17	69 ± 5	88 ± 15	151 ± 21

### Determination of the cytotoxicity of cisplatin-conjugated magnetic nanoparticles

**Figure 2:**
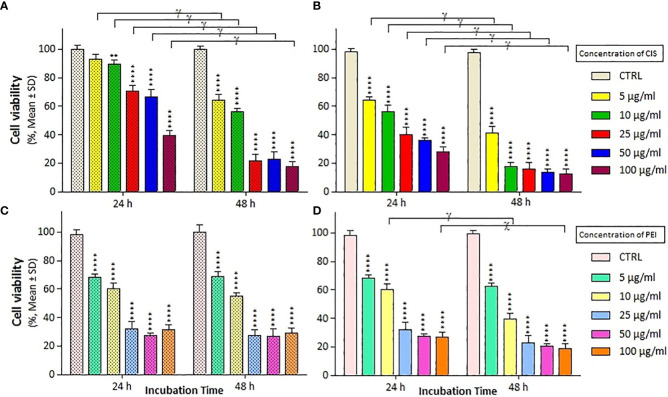
The cell viability results of **(A, C)** A2780/CP and **(B, D)** A2780 cells treated with cisplatin (CIS) and polyethylenimine (PEI) at the same different concentrations (5, 10, 25, 50, and 100 µg/ml) for two exposure times (24 and 48 h), respectively. Cell viability values were determined by MTT assay and microplate reader, and results are expressed as the percentage of viable cells. Data are shown as mean ± SD (n = 3). **P<0.01; ****P<0.0001 show significant differences relative to unexposed cells (CTRL), and letters (χ,P<0.001; γ,P<0.0001) show significant differences between treated cells (6 × 2 factorial ANOVA flowed by *post-hoc* Newman–Keuls multiple comparison tests).

We assessed the viability of A2780/CP and A2780 cells with different treatments at 24 and 48 h using MTT staining and a microplate reader. As shown in **Figures 2A, B**, treatment with CIS decreased the viability of A2780 cells more than that of A2780/CP cells, with a reduction of around 30% at concentration of 5, 10 and 25 µg/ml after 24 h, and around 20% and 10% at concentration of 50 and 100 µg/ml, respectively. After 48 h, the reduction in viability was more pronounced, with decreases of around 20% and 40% at concentrations of 5 and 10 µg/ml, respectively. Treatment with PEI significantly decreased the viability of both cell lines compared to untreated cells at both time points, with no significant difference between the two cell lines. In the A2780 cell line, after 24 h, the rate of cell death was approximately 40%, 60%, and 80% in the CIS, PM/C, and PMS treatments, respectively, and this effect was more pronounced after 48 h.

**Figure 3:**
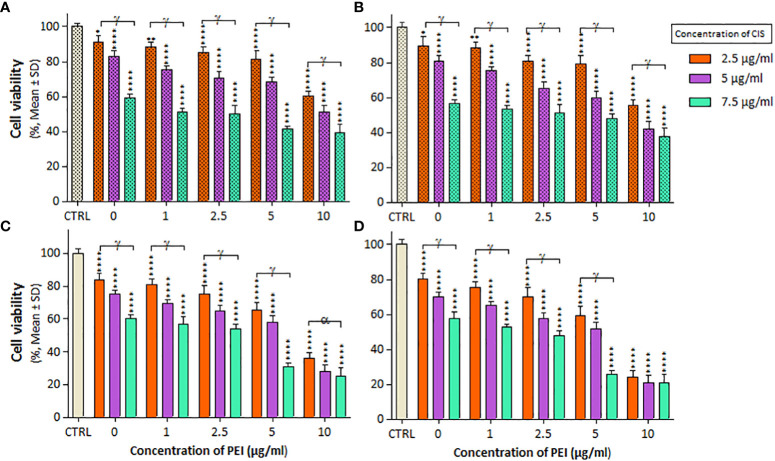
The cell viability results of **(A, B)** A2780/CP and **(C, D)** A2780 cells treated with PC binary complexes involve different concentrations of 1, 2/5, 5, and 10 µg/ml polyethylenimine (PEI), and 2.5, 5 and 7.5 µg/ml cisplatin (CIS) for two exposure times (24 and 48 h), respectively. Cell viability values were determined by MTT assay and microplate reader, and results are expressed as the percentage of viable cells. Data are shown as mean ± SD (n = 3). *P<0.05; **P<0.01; ****P<0.0001 show significant differences relative to unexposed cells (CTRL), and letters (α,P<0.05; γ,P<0.0001) show significant differences between treated cells (5 × 4 factorial ANOVA flowed by *post-hoc* Newman–Keuls multiple comparison tests).

The combined effects of CIS and PEI are shown in **Figure 3**, where the PC binary complex significantly increased cytotoxicity at different concentrations in both cell lines. Increasing PEI concentration from 2.5 μg/mL onwards at constant concentrations of CIS (2.5, 5, and 7.5 µg/mL) resulted in a decrease in viability was observed in both cell lines. A2780 cells showed a greater decrease in viability than A2780/CP cells, with reductions of around 20%, 10% and 15% at the concentration (2.5, 5 and 7.5 µg/ml) of CIS, respectively, and a constant concentration of 10 µg/mL of PEI after 24 h. Similarly, reductions of around 20%, 10%, and 20% at concentrations of 2.5, 5, and 7.5 µg/ml of CIS and a constant concentration of 5 µg/mL of PEI, and around 40%, 20%, and 15% at concentrations of 2.5, 5, and 7.5 µg/ml of CIS and a constant concentration of 10 µg/mL of PEI after 48 h, respectively.

**Figure 4:**
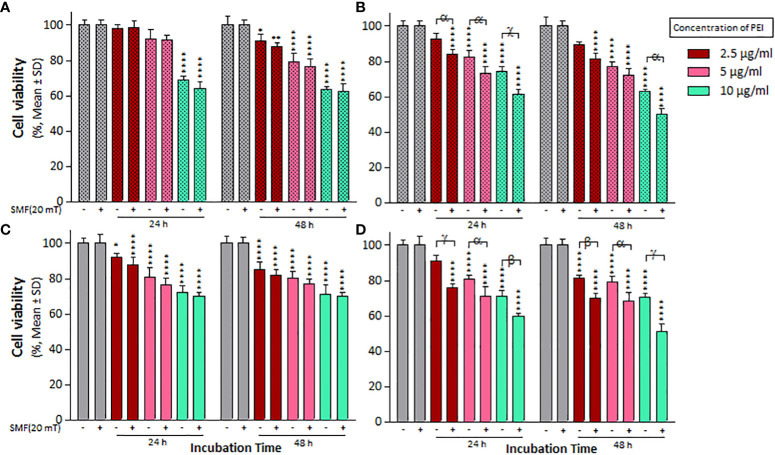
The cell viability results of SMF (20 mT) exposure on **(A)** A2780/CP and **(C)** A2780 cells treated with different concentrations of 2.5, 5, and 10 µg/ml polyethylenimine (PEI), and **(B)** A2780/CP and **(D)** A2780 cells exposed to combinations of varying concentrations of 2.5, 5, and 10 µg/ml polyethylenimine (PEI) and 1 µg/ml magnetite nanoparticles (MNPs) for two exposure times (24 and 48 h), respectively. Cell viability value was determined by MTT assay and microplate reader, and results are expressed as the percentage of viable cells. Data are shown as mean ± SD (n = 3). *P<0.05; ** P<0.01; ****P<0.0001 show significant differences relative to unexposed cells (CTRL), and letters (α,P<0.05; β,P<0.01 χ,P<0.001; γ,P<0.0001) show significant differences between treated cells (4 × 2 × 2 factorial ANOVA flowed by *post-hoc* Newman–Keuls multiple comparison tests).

The effect of 20 mT SMF exposure on cell viability and IC_50_ values was evaluated after treatments with the PEI and PM binary complex, as indicated in **Figure 4** and **Table 2**. Notably, the cell viability and IC_50_ significantly decreased in A2780 cells treated with PEI compared to A2780/CP cells, particularly in the presence of SMF, at both times (**Figures 4A, C**). Additionally, both cell lines exhibited a substantial reduction in cell viability and IC_50_ values when treated with MNPs within the PM complexes under SMF exposure after 24 h (**Figurse 4B, D**). Furthermore, the combined application of MNPs and PEI resulted in a more pronounced decrease in the viability and IC_50_ values of A2780 cells than A2780/CP cells after 48 h. Specifically, the results revealed that MNPs decreased the cell viability of A2780/CP cells compared to untreated cells only in the presence of SMF, with reductions of approximately 10% observed at the concentration of 2.5, 5 and 10 µg/ml of PEI after 24 h, and a reduction of 10% observed at a concentration 10 µg/ml of PEI after 48 h. Similarly, in A2780 cells, reductions of around 20%, 10%, and 15% were observed at concentrations of 2.5, 5, and 10 µg/ml of PEI after 24 h, respectively. After 48 h, reductions of approximately 15%, 10%, and 20% were observed at concentrations of 2.5, 5, and 10 µg/ml of PEI, respectively.

**Table 2:** The inhibitory concentration 50% (IC_50_) of CIS, PEI, PC, PM, PM/C, and PMC treatments were calculated in A2780/CP and A2780 cells in presence and absence of 20 mT static magnetic field (SMF).

Cells	CIS (µg/ml)		PEI (µg/ml)		PC (µg/ml)		PM (µg/ml)		PM/C (w/w)		PMC (w/w)	
**A2780/CP (24 h)**	15.7±1.1	15.2±2.3	28.4±1.3	27.4± 2.2	14±1.1	13.2±3.1	25.9±2.3	14.5±2.2	5.9±1.3	4.7±0.8	4.8±0.9	3.9±0.9
**A2780 (24 h)**	11.1±0.6	10.1±3.2	23.8±3.1	20.2±0.9	8.5±0.5	7.9±2.9	23.6±3.4	12.5±3.1	5.1±0.8	4±1.1	3.9±0.7	3.2±0.7
**A2780/CP (48 h)**	14±0.8	13.1±0.3	21.9±2.4	18.9±0.9	11±1.1	10.1±0.8	17.3±1.7	11±0.9	3.7±0.8	2.7±0.2	2.7±0.2	1.6±0.2
**A2780 (48 h)**	10.6±1.2	9.5±0.4	18.4±1.6	15.9±0.3	8.4±0.6	7.3±0.4	19.8±1.9	9.3±1.8	3.2±0.3	2.3±0.3	1.6±0.3	0.6±0.2
**SMF (20 mT)**	**-**	**+**	**-**	**+**	**-**	**+**	**-**	**+**	**-**	**+**	**-**	**+**

**Figure 5:**
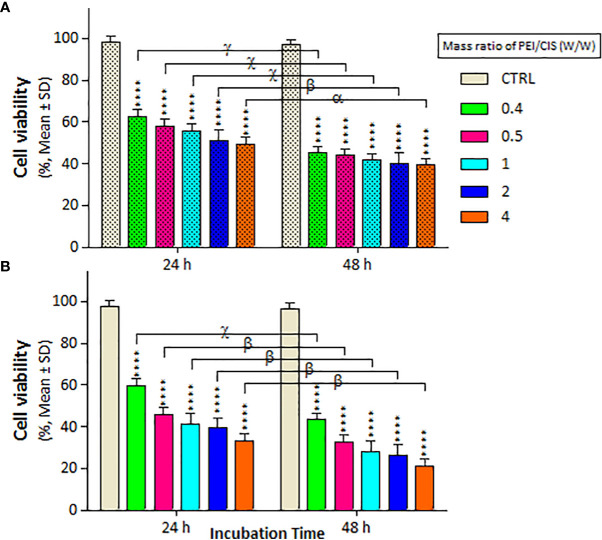
The cell viability results of **(A)** A2780/CP and **(B)** A2780 cells treated with three-component magnetic nanocomplex (PEI-MNP-CIS (PMC)) including different mass ratios (0, 0.4, 0.5, 1, 2, and 4 w/w) of polyethylenimine (PEI) compared to cisplatin (CIS) at same concentration 1 µg/ml of Fe_3_O_4_ magnetite nanoparticles (MNPs) for two exposure times (24 and 48 h), respectively. Cell viability values were determined by MTT assay and microplate reader, and results are expressed as the percentage of viable cells. Data are shown as mean ± SD (n = 3). ****P<0.0001 show significant differences relative to unexposed cells (CTRL), and letters (α,P<0.05; β,P<0.01; χ,P<0.001; γ,P<0.0001) show significant differences between treated cells (6 × 2 factorial ANOVA flowed by *post-hoc* Newman–Keuls multiple comparison tests).

**Figure 6:**
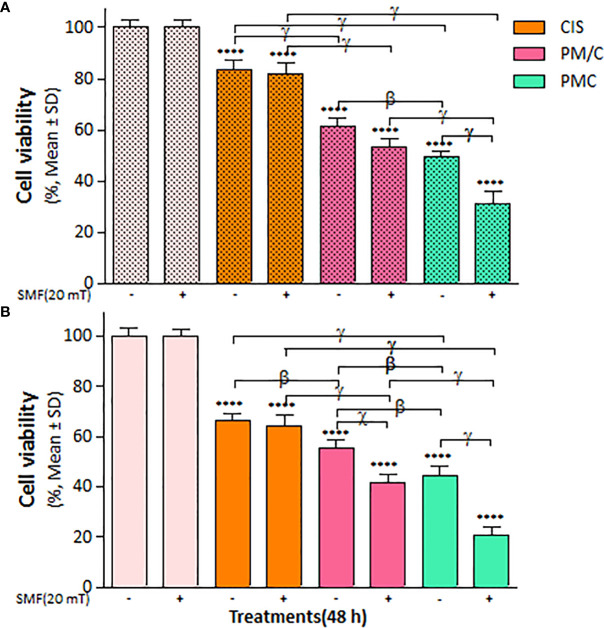
The cell viability results of **(A)** A2780/CP and **(B)** A2780 cells treated with (PEI-MNPs/CIS (PM/C)) consist of cisplatin (CIS)-treatment (2.5 μg/ml) after 1 h of PEI-MNPs (PM) binary complex treatment at concentrations of 1 μg/ml polyethylenimine (PEI) and 1 μg/ml Fe_3_O_4_ magnetite nanoparticles (MNPs), and also three-component magnetic nanocomplex (PEI-MNP-CIS (PMC)) at the same concentrations of PM/C in presence and absence of 20 mT static magnetic field (SMF) for 48 (h) Cell viability values were determined by MTT assay and microplate reader, and results are expressed as the percentage of viable cells. Data are shown as mean ± SD (n = 3). ****P<0.0001 show significant differences relative to unexposed cells (CTRL), and letters (β,P<0.01; χ,P<0.001; γ,P<0.0001) show significant differences between treated cells (2 × 2 × 2 × 2 factorial ANOVA flowed by *post-hoc* Newman–Keuls multiple comparison tests).

The mass of PEI used in the nanocomplexes is an important parameter. As shown in **Figure 5**, the mass ratio of PEI/CIS decreased the viability of A2780 cells more than A2780/CP cells in the PMC nanocomplex at different concentrations of PEI and a constant concentration of 2.5 µg/mL CIS and 1 µg/mL MNPs. Indeed, PEI boosted CIS toxicity in both cell lines, particularly in A2780 cells at 48 h. Moreover, in **Figure 6**, the results indicate that the cytotoxicity effect of PM/C and PMC, at a mass ratio of 0.4 and including 2.5 µg/mL of CIS, 1 µg/mL of PEI, and 1 µg/mL of MNPs, was enhanced in the presence of a 20 mT SMF in both cell lines, especially in A2780 cells. In the A2780/CP cell line, the rate of cell death was around 20%, 45%, and 70% in the CIS, PM/C, and PMS treatments, respectively, in the presence of SMF. The same trend was observed in the A2780 cell line, with rates of cell death of approximately 40%, 60%, and 80% in the CIS, PM/C, and PMS treatments, respectively, in the presence of SMF. Furthermore, the effects of PMC on cell viability were greater than those of the PM/C complexes in the presence and absence of SMF. The IC_50_ values of CIS, PEI, PC, PM, PM/C, and PMC were calculated for the A2780/CP and A2780 cells at 24 and 48 h (**Table 2**). These results suggest that the PMC complexes are more effective than the PM/C complexes in mediating the cytotoxic effects of CIS.

### Cisplatin increases intracellular ROS production

**Figure 7:**
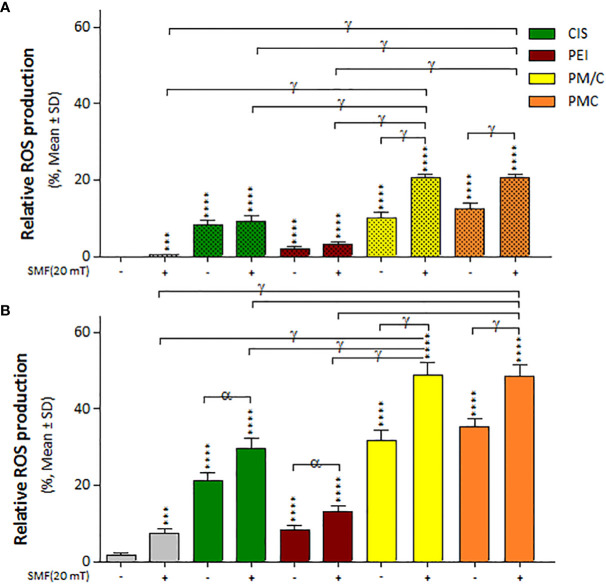
The intracellular ROS generation of **(A)** A2780/CP and **(B)** A2780 cells treated with 1 μg/ml of polyethylenimine (PEI), 2.5 μg/ml of cisplatin (CIS), and PM/C and PMC three-component magnetic nanocomplexes at same concentrations (1 μg/ml PEI, 1 μg/ml MNPs and 2.5 μg/ml CIS) in presence and absence of 20 mT static magnetic field (SMF) for 48 (h) Cells were collected, and the ROS generation was evaluated using oxidized DCFDA and Flow-Cytometry analysis. Data are shown as mean ± SD (n = 3). ***P<0.001; ****P<0.0001 show significant differences relative to unexposed cells (CTRL), and letters (α,P<0.05; γ,P<0.0001) show significant differences between treated cells (2 × 2 × 2 × 2 × 2 factorial ANOVA flowed by *post-hoc* Newman–Keuls multiple comparison tests).

We measured intracellular ROS accumulation in the A2780/CP and A2780 cells in the presence and absence of a 20 mT SMF after treatment with CIS, PEI, and ternary complexes (PM/C and PMC) at the same concentration for 48 h using the fluorescent probe DCFH-DA and flow cytometry (**Supplementary Figure S1**). As shown in **Figure 7**, ROS levels significantly increased in all treatments compared to untreated cells. In general, A2780 cells exhibited higher ROS levels than A2780/CP cells in all treatments than A2780/CP cells in all treatments. Moreover, the PM/C and PMC complexes equally increased ROS production to a greater extent than the SMF, CIS and PEI-treated cells in both cell lines. ROS levels significantly increased in PM/C and PMC complexes in the presence of 20 mT SMF in the both cell lines. Additionally, ROS levels increased in the CIS and PEI-treated A2780 cells in the presence of 20 mT SMF compared to absence of 20 mT SMF.

### Cisplatin induces S and G2/M phase cell cycle arrest

**Figure 8:**
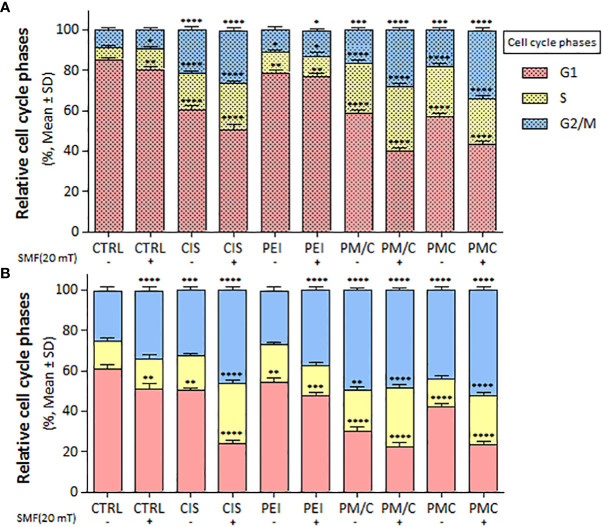
The cell cycle arrest of **(A)** A2780/CP and **(B)** A2780 cells treated with 1 μg/ml of polyethylenimine (PEI), 2.5 μg/ml of cisplatin (CIS), and PM/C and PMC three-component magnetic nanocomplexes at same concentrations (1 μg/ml PEI, 1 μg/ml MNPs and 2.5 μg/ml CIS) in presence and absence of 20 mT static magnetic field (SMF) for 48 (h) Cells were analyzed for cell cycle distribution through a PI flow cytometry kit. The plot shows cells in G1, S and G2/M phases. Data are shown as mean ± SD (n = 3). *P<0.05; **P<0.01 ***P<0.001; ****P<0.0001 show significant differences relative to unexposed cells (CTRL). (2 × 2 × 2 × 2 × 2 factorial ANOVA flowed by *post-hoc* Newman–Keuls multiple comparison tests).

We evaluated the cell cycle distribution of A2780/CP and A2780 cells in the presence and absence of 20 mT SMF after treatment with CIS, PEI, and ternary complexes (PM/C and PMC) at the same concentration of 2.5 μM CIS, 1 μM PEI, and 1 μM MNP for 48 h using PI staining and flow cytometry (**Supplementary Figure S2**). As shown in **Figure 8**, the application of SMF led to the S phase arrest of A2780/CP cells and G2/M phase arrest of A2780 cells. In the presence and absence of a 20 mT SMF, CIS triggered S and G2/M phase arrest of A2780/CP cell lines. Similarly, in the presence of 20 mT SMF, CIS induced S and G2/M phase arrest of A2780 cell lines. Furthermore, after PEI treatment, a higher proportion of SMF-treated A2780 cells than SMF-treated A2780/CP cells was arrested in G2/M. In addition, PMC and PM/C treatments induced S and G2/M phases in the presence of SMF in both cell lines. In the A2780/CP cells, PM/C and PMC complexes induced G2/M phase arrest in the presence of SMF compared to absent of SMF. In the A2780 cells, PM/C and PMC complexes induced S phase arrest in the presence of SMF compared to absent of SMF.

### Cisplatin promotes apoptosis in cancer cells

**Figure 9:**
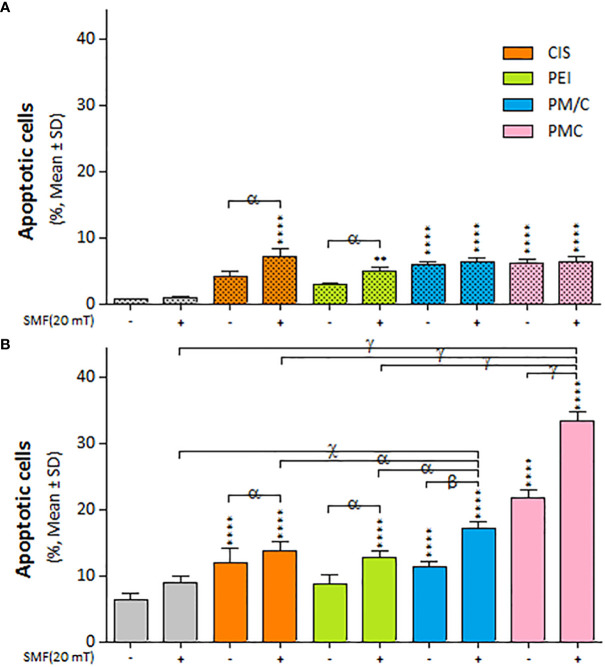
The apoptosis cell death of **(A)** A2780/CP and **(B)** A2780 cells treated with 1 μg/ml of polyethylenimine (PEI), 2.5 μg/ml of cisplatin (CIS), and PM/C and PMC three-component magnetic nanocomplexes at same concentrations (1 μg/ml PEI, 1 μg/ml MNPs and 2.5 μg/ml CIS) in presence and absence of 20 mT static magnetic field (SMF) for 48 (h) Cells were collected and labeled with annexin V/PI for flow cytometric analysis. The bar graphs represented the mean values of the apoptotic cells (Q2+Q3). Data are shown as mean ± SD (n = 3). **P<0.01; ****P<0.0001 show significant differences relative to unexposed cells (CTRL), and letters (α,P<0.05; β,P<0.01 χ,P<0.001; γ,P<0.0001) show significant differences between treated cells (2 × 2 × 2 × 2 × 2 factorial ANOVA flowed by *post-hoc* Newman–Keuls multiple comparison tests).

We investigated the apoptosis rate of A2780/CP and A2780 cells in the presence and absence of SMF (20 mT) after treatment with CIS, PEI, and PM/C and PMC ternary complexes simultaneously for 48 h using annexin/PI and flow cytometry (**Supplementary Figure S3**). As illustrated in **Figure 9**, exposure to SMF modulated the rate of apoptotic cell death in the A2780 cells, but the effect was less pronounced in the A2780/CP cells. Applying SMF increased the levels of apoptosis after CIS and PEI treatments in either cell line. In the A2780 cells, the apoptosis rate significantly increased after the PMC and PM/C treatments compared to SMF, CIS and PEI treatments alone. Treatment with the PMC ternary complex resulted in a higher level of apoptosis than the other treatments in the A2780 cell line. Indeed, PMC was more effective than PM/C at inducing apoptosis in the A2780 cell line. Application of SMF increased the levels of apoptosis after PM/C and PMC treatments in the A2780 cell line, while it did not cause any changes in the A2780/CP cell lines.

## Discussion

This study aimed to investigate the effects of PEI-based magnetic nanocomplexes on ovarian cancer cells in the presence of SMF and the likely mechanisms of these effects. Cancer is caused by gene mutations and changes in cell physiology and function. Here, the ternary CIS complexes increased cytotoxicity and cell accumulation in the S and G2/M phases of the cell cycle, as well as ROS production and apoptosis. Polymeric nanocomplexes have been extensively researched to increase the cytotoxicity and efficiency of delivery systems, enhancing drug toxicity and overcoming chemotherapy-drug resistance. Tseng et al. reported that CIS-incorporated gelatin nanocomplexes can be used for cancer chemotherapy (49). Furthermore, Lipid and polymer-based nanoparticle siRNA delivery systems have also been developed by Mainini and Eccles for cancer therapy (50).

Polymeric nanoparticles synthesized based on host–guest interactions between β-cyclodextrin and benzimidazole have been used for liver cancer-targeted therapy, demonstrating a remarkable ability to induce cell apoptosis (51). Additionally, Lim et al. used redox-responsive polymeric nanocomplexes to deliver cytotoxic proteins and chemotherapeutics. They synthesized nanocomplexes, including PEI cross-linked with oxaliplatin (IV) pro-drug, to generate ROS in the cell, which can stimulate controlled drug release and affect protein reactivity (52). In this study, we synthesized a noncomplex that included PEI cross-linked by CIS and MNPs as ternary complexes (PMC), which were characterized by FTIR (**Figure 1**) and DLS (**Table 1**). The developed complex enhanced CIS cytotoxicity and potentially overcame drug resistance. As shown in **Figure 2**, the application of CIS and PEI decreased the viability of A2780 cells more than that of A2780/CP cells. Cytotoxicity increased in the PC (**Figure 3**) and PM (**Figure 4**) binary complexes, as well as in the PMC ternary complex in both cell lines, especially A2780 cell lines (**Figure 6**).

CIS causes cell death by binding and disrupts the nuclear (n)DNA and mitochondrial DNA (mt)DNA, inhibiting the transcription and replication of nDNA and mtDNA (53). Cationic polymers bind to negatively charged proteins in the cells, such as heat shock proteins and glutathione S-transferases, which are involved in apoptosis. This is the most important mechanism of toxicity by PEI polymers and enhances CIS cytotoxicity in the PC, PM, and PMC complexes (54, 55). Recently, Karimi et al. reported the use of CoFe_2_O_4_/MNPs as effective carriers for the delivery of anticancer drugs such as epirubicin (56).

Our study showed that ROS production significantly increased under all treatment conditions (**Figure 7**). In both cell lines, treatment with PMC and PM/C complexes enhanced ROS production more than treatments with SMF, CIS and PEI did. In general, the level of ROS produced in A2780 cells was higher than in A2780/CP cells under all treatments. Free radicals are essential to maintain low levels of physiological and proliferation processes in the cell. However, high ROS accumulation leads to oxidative damage of cell components and cell death (15, 22). Platinum in CIS induces ROS production, leading to lesions in DNA, proteins and lipids, and ultimately promoting apoptosis (57). It is possible that the PEI polymers present in the PMC and PM/C complexes may inhibit the activity of various antioxidant enzymes, including glutathione S-transferases, which are responsible for scavenging ROS. This disruption of antioxidant enzyme activity may cause an increase in ROS content within the cells, leading to apoptosis.

CIS induced A2780/CP to accumulate in the S and G2/M phases of the cell cycle in presence and absence of 20 mT SMF, while CIS induced S and G2/M phase arrest of A2780 cell lines only in presence of 20 mT SMF. Notably, G2/M arrest after PEI treatment was more evident in SMF-treated A2780 cells than in A2780/CP cells. Furthermore, PMC and PM/C treatments induced S and G2/M phase cell cycle arrest by decreasing the cell population in the G0/G1 phase in the presence of SMF in both cell lines. In the A2780/CP cells, PM/C and PMC complexes induced G2/M phases in the presence of SMF compared to absent of SMF. In A2780 cells, PM/C and PMC complexes induced S phases in the presence of SMF compared to the absent of SMF (**Figure 8**). Elevated ROS levels can lead to oxidative damage of nucleotides in the nucleotide pool and DNA molecules. Free radicals cause various types of damage, including single- or double-stranded breaks in DNA, and eventually activate proteins such as the phosphorylated form of checkpoint kinase ataxia telangiectasia mutated (ATM) in the cells. ATM phosphorylates Cdc25, p53, and E2F1, initiating the repair process or apoptosis of the damaged cells, whereby their inactivation induces G2/M phase arrest (58–61).

**Figure 9** illustrates the impact of SMF application (within each treatment group) on the rate of apoptotic cell death in A2780 and A2780/CP cells. The results indicate that the effect of SMF was less pronounced in A2780/CP cells compared to A2780 cells. The apoptosis rate was significantly higher in PMC and PM/C treatments compared to SMF, CIS and PEI treatments in the A2780 cell lines. Notably, treatment with the PMC ternary complex resulted in higher levels of apoptosis in A2780 cells compared to other treatments, and PMC was more effective than PM/C at inducing apoptosis. The PMC complexes are likely to trigger apoptosis through two mechanisms - firstly, the inhibitory effect of PEI on negatively charged proteins, and secondly, the conjugation of CIS to PM, which enhances the efficiency of drug transfer by PMC. Conversely, the effects mediated by the PM/C complexes rely only on the former mechanism. Additionally, SMF application may influences cell functions during MNPs treatment through mechanical forces that facilitate the penetration of the cell membrane and promote apoptosis in the A2780/CP cell lines (62).

Our results in **Figure 6** indicate that the rate of cell death in A2780/CP cell lines was around 20%, 45%, and 70% in the CIS, PM/C, and PMS treatments, respectively in the presence of SMF. Similarly, in the A2780 cell lines, the rate of cell death was approximately 40%, 60%, and 80% in the CIS, PM/C, and PMS treatments, respectively. Additionally, as shown in **Figure 9**, the rate of cell apoptosis in the presence of SMF was approximately 10% in the A2780/CP cell lines and 15%, 20%, and 30% in the A2780 cell lines in the CIS, PM/C, and PMS treatments, respectively. Furthermore, **Supplementary Figure S3** showed the rate of necrosis (Q1) in the presence of SMF was around 4.3%, 4.7%, and 8.6% in the A2780/CP cells and 5.2%, 6.1%, and 3.4% in the A2780 cells in the CIS, PM/C, and PMS treatments, respectively. Therefore, it is plausible that in addition to apoptosis and necrosis, other forms of cell death such as ferroptosis, cuproptosis, or disulfidoptosis may have occurred.

Ferroptosis is a type of regulated cell death that is dependent on iron and ROS, as well as its characteristic lipid peroxidation. It is morphologically and biochemically distinct and disparate from other processes of cell death. This process occurs as a result of a coordinated interplay between iron availability, ROS generation, glutamate excess, and cysteine depletion. The magnetite (Fe3O4) nanoparticles used in our study may have induced ferroptosis by supplying iron. Cysteine depletion, which is known to trigger ferroptosis, leads to the degradation of ferritin via ferritinophagy, a type of autophagy mediated by nuclear receptor activator 4 (NCOA4) (63). NCOA4 regulates iron homeostasis and produces ROS in cells, thus playing a crucial role in inducing ferroptosis (64). In addition, cuproptosis is a unique form of cell death that is dependent on copper and distinct from other types of cell death (65). In a previous study, the association between 10 cuprotosis-associated genes (CAGs) and HNSC was investigated using multi-omics public data. The expression of these CAGs was found to be correlated with the sensitivity of cancer cells to multiple drugs, including cisplatin and docetaxel (66). Therefore, it is possible that cuproptosis occurred in the A2780/CP and 2780 cells and influenced the sensitivity of cisplatin by increasing ROS levels, ultimately leading to an increase in the rate of cell death.

It is possible that during the application of PM/C complexes, the PEI polymer and MNPs initially entered the cell without CIS, and the PEI polymer had a sufficient opportunity to inhibit negatively charged proteins. This neutralization of proteins may have contributed to a decrease in drug resistance in A2780/CP cells. Conversely, in treatments with the PMC complexes, all components were entered into the cells simultaneously, resulting in reduced efficiency of PEI compared to PM/C complexes. Most proteins involved in CIS resistance have an isoelectric pH of less than ~7.2 and carry a negative charge at neutral pH, making them attractive targets for PEI binding. Therefore, the abundance of negatively charged proteins in A2780 cells may be relatively low. This is reflected by the less significant changes in the IC50 rate after CIS treatment in A2780 cells compared to A2780/CP cells, as shown in **Table 2**. Additionally, **Table 3** indicates that the net charge of the proteins involved in pre-target resistance is negative. One such protein is the glutathione S-transferase enzyme, the expression of which has been associated with increased resistance to CIS in previous studies (68, 69). The net charge and isoelectric pH of the enzyme are 3 and 5.3, respectively. Glutathione S-transferase enzymes are essential scavengers of ROS, and their function leads to ROS accumulation, G2/M phase arrest, and apoptosis (54, 70). PEI can bind to these negatively charged enzymes as well as other biological molecules with negative charge, such as mRNA (55). Therefore, proteins highly linked to resistance to CIS may have an increased cytoplasmatic mRNA content, and PEI may inhibit ROS scavengers in the pre-target mechanism and the translation of proteins involved in drug resistance.

**Table 3:** The mechanisms, functions, isoelectric point (pI), and net charge of enzymes are involved in the drug resistance of cancer cells.

Enzyme	Mechanism	Function	Isoelectric point (pI)	Net charge
**Glutathione synthetase (GSS)**	Pre-target resistance	GSH scavenges electrophiles and ROS (16)	**3.3**	**-1**
**Glutathione- S-transferase (GST)**	Pre-target resistance	Inactivation of cisplatin through conjugation to GSH (15)	**5.3**	**-3**
**Gamma-glutamylcysteine synthetase (γ-GCS)**	Pre-target resistance	Conjugates GSH to cisplatin and facilitates its excretion (67)	**5.7**	**-13**
**ATPase copper transporting beta (ATP7B)**	Pre-target resistance	Involved in the export of copper and cisplatin out of the cells (8, 11)	**6.2**	**-11.9**
**ATPase copper transporting alpha (ATP7A)**	Pre-target resistance	Involved in the export of copper and cisplatin out of the cells (11)	**5.9**	**-19.9**

The high buffering capacity of PEI and its ability to induce endosomal escape can trigger endocytosis-mediated delivery, which is crucial for effective drug delivery. Endosomal escape allows a carrier to enter the nucleus before it is destroyed by endosomes and intracellular lysosomes (71, 72). Therefore, PMC complexes may appear less abundant in the cell cytoplasm. and may not be easily recognized by lysosomes or multi-drug resistance mechanisms. They are also less exposed to antioxidants, such as glutathione and metallothionein, which can reduce the effectiveness of CIS. In conclusion, PEI-based magnetic nanocomplexes may represent a promising strategy for enhancing CIS cytotoxicity and overcoming drug resistance in cancer cells.

## Data availability statement

The original contributions presented in the study are included in the article/**Supplementary Material**. Further inquiries can be directed to the corresponding author.

## Ethics statement

Ethical approval was not required for the studies on animals in accordance with the local legislation and institutional requirements because only commercially available established cell lines were used.

## Author contributions

All authors contributed to the conception and design of the study. Preparation of material, data collection, and analysis were performed by FA, BH-V, and MS. The first draft was written by FA, and all authors commented on different versions of the manuscript. All authors contributed to the article and approved the submitted version.

## References

[1] Hashim S Shukur R Jaafer H Jaafer H Al-Rawaq K Alshewered A. The assessment of Malignant ovarian tumors in Baghdadian women. Prensa Med Argent (2020) 106:173. doi: 10.47275/0032-745X-173

[2] Perrone MG Luisi O De Grassi A Ferorelli S Cormio G Scilimati A. Translational Theragnosis of Ovarian Cancer: where do we stand? Curr Medicinal Chem (2020) 27:5675–715. doi: 10.2174/0929867326666190816232330 31419925

[3] Mikuš M Benco N Matak L Planinić P Ćorić M Lovrić H. Fertility-sparing surgery for patients with Malignant ovarian germ cell tumors: 10 years of clinical experience from a tertiary referral center. Arch Gynecol Obstetrics (2020) 301:1227–33. doi: 10.1007/s00404-020-05522-5 32253553

[4] Fuertes M Castilla J Alonso C Prez J. Cisplatin biochemical mechanism of action: from cytotoxicity to induction of cell death through interconnections between apoptotic and necrotic pathways. Curr medicinal Chem (2003) 10:257–66. doi: 10.2174/0929867033368484 12570712

[5] Bertolini G Roz L Perego P Tortoreto M Fontanella E Gatti L. Highly tumorigenic lung cancer CD133+ cells display stem-like features and are spared by cisplatin treatment. Proc Natl Acad Sci (2009) 106:16281–6. doi: 10.1073/pnas.0905653106 PMC2741477 19805294

[6] Dasari S Tchounwou PB. Cisplatin in cancer therapy: molecular mechanisms of action. Eur J Pharmacol (2014) 740:364–78. doi: 10.1016/j.ejphar.2014.07.025 PMC4146684 25058905

[7] Baruah H Rector CL Monnier SM Bierbach U. Mechanism of action of non-cisplatin type DNA-targeted platinum anticancer agents: DNA interactions of novel acridinylthioureas and their platinum conjugates. Biochem Pharmacol (2002) 64:191–200. doi: 10.1016/S0006-2952(02)01107-3 12123739

[8] Mcmullen M Madariaga A Lheureux S. New approaches for targeting platinum-resistant ovarian cancer. Semin Cancer Bio (2021) 77:167–81. doi: 10.1016/j.semcancer.2020.08.013 32871277

[9] Samimi G Safaei R Katano K Holzer AK Rochdi M Tomioka M. Increased expression of the copper efflux transporter ATP7A mediates resistance to cisplatin, carboplatin, and oxaliplatin in ovarian cancer cells. Clin Cancer Res (2004) 10:4661–9. doi: 10.1158/1078-0432.CCR-04-0137 15269138

[10] Pabla N Murphy RF Liu K Dong Z. The copper transporter Ctr1 contributes to cisplatin uptake by renal tubular cells during cisplatin nephrotoxicity. Am J Physiology-Renal Physiol (2009) 296:F505–11. doi: 10.1152/ajprenal.90545.2008 PMC2660190 19144690

[11] Galluzzi L Senovilla L Vitale I Michels J Martins I Kepp O. Molecular mechanisms of cisplatin resistance. Oncogene (2012) 31:1869–83. doi: 10.1038/onc.2011.384 21892204

[12] Borst P Evers R Kool M Wijnholds J. A family of drug transporters: the multidrug resistance-associated proteins. J Natl Cancer Institute (2000) 92:1295–302. doi: 10.1093/jnci/92.16.1295 10944550

[13] Nies AT König J Pfannschmidt M Klar E Hofmann WJ Keppler D. Expression of the multidrug resistance proteins MRP2 and MRP3 in human hepatocellular carcinoma. Int J Cancer (2001) 94:492–9. doi: 10.1002/ijc.1498 11745434

[14] Materna V Liedert B Thomale J Lage H. Protection of platinum–DNA adduct formation and reversal of cisplatin resistance by anti-MRP2 hammerhead ribozymes in human cancer cells. Int J Cancer (2005) 115:393–402. doi: 10.1002/ijc.20899 15688364

[15] Rabik CA Dolan ME. Molecular mechanisms of resistance and toxicity associated with platinating agents. Cancer Treat Rev (2007) 33:9–23. doi: 10.1016/j.ctrv.2006.09.006 17084534 PMC1855222

[16] Korita PV Wakai T Shirai Y Matsuda Y SAKATA J Takamura M. Multidrug resistance-associated protein 2 determines the efficacy of cisplatin in patients with hepatocellular carcinoma. Oncol Rep (2010) 23:965–72. doi: 10.3892/or_00000721 20204280

[17] Bedford P Walker MC Sharma HL Perera A Mcauliffe CA Masters JR. Factors influencing the sensitivity of two human bladder carcinoma cell lines to cis-diamminedichloro-platinum (II). Chemico-biological Interact (1987) 61:1–15. doi: 10.1016/0009-2797(87)90015-9 3815585

[18] Meijer C Mulder NH Timmer-Bosscha H Sluiter WJ Meersma GJ de Vries EG. Relationship of cellular glutathione to the cytotoxicity and resistance of seven platinum compounds. Cancer Res (1992) 52:6885–9. 1458477

[19] Jansen BA Brouwer J Reedijk J. Glutathione induces cellular resistance against cationic dinuclear platinum anticancer drugs. J inorganic Biochem (2002) 89:197–202. doi: 10.1016/S0162-0134(02)00381-1 12062123

[20] Krieger ML Eckstein N Schneider V Koch M Royer H-D Jaehde U. Overcoming cisplatin resistance of ovarian cancer cells by targeted liposomes in *vitro* . Int J pharmaceutics (2010) 389:10–7. doi: 10.1016/j.ijpharm.2009.12.061 20060458

[21] Kuo MT Fu S Savaraj N Chen HH. Role of the human high-affinity copper transporter in copper homeostasis regulation and cisplatin sensitivity in cancer chemotherapy. Cancer Res (2012) 72:4616–21. doi: 10.1158/0008-5472.CAN-12-0888 PMC3445735 22962276

[22] Hajipour Verdom B Abdolmaleki P Behmanesh M. The static magnetic field remotely boosts the efficiency of doxorubicin through modulating ROS behaviors. Sci Rep (2018) 8:990. doi: 10.1038/s41598-018-19247-8 29343746 PMC5772617

[23] Li YQ Yin JY Liu ZQ Li XP. Copper efflux transporters ATP7A and ATP7B: Novel biomarkers for platinum drug resistance and targets for therapy. IUBMB Life (2018) 70:183–91. doi: 10.1002/iub.1722 29394468

[24] Ghasemi M Sivaloganathan S. A computational study of combination HIFU–chemotherapy as a potential means of overcoming cancer drug resistance. Math Biosci (2020) 329:108456. doi: 10.1016/j.mbs.2020.108456 32841615

[25] Jiang W Xia J Xie S Zou R Pan S Wang Z-W. Long non-coding RNAs as a determinant of cancer drug resistance: Towards the overcoming of chemoresistance via modulation of lncRNAs. Drug Resistance Updates (2020) 50:100683. doi: 10.1016/j.drup.2020.100683 32146422

[26] Zhang Z Xie Z Sun G Yang P Li J Yang H. Reversing drug resistance of cisplatin by hsp90 inhibitors in human ovarian cancer cells. Int J Clin Exp Med (2015) 8:6687. doi: 1997-2010.doi:10.2147/IJN.S193170 26221207 PMC4509152

[27] Ai Z Lu Y Qiu S Fan Z. Overcoming cisplatin resistance of ovarian cancer cells by targeting HIF-1-regulated cancer metabolism. Cancer Lett (2016) 373:36–44. doi: 10.1016/j.canlet.2016.01.009 26801746 PMC4769873

[28] Hu C-MJ Zhang L. Nanoparticle-based combination therapy toward overcoming drug resistance in cancer. Biochem Pharmacol (2012) 83:1104–11. doi: 10.1016/j.bcp.2012.01.008 22285912

[29] Wang H Huang Y. Combination therapy based on nano codelivery for overcoming cancer drug resistance. Med Drug Discovery (2020) 6:100024. doi: 10.1016/j.medidd.2020.100024

[30] Chang P-Y Peng S-F Lee C-Y Lu C-C Tsai S-C Shieh T-M. Curcumin-loaded nanoparticles induce apoptotic cell death through regulation of the function of MDR1 and reactive oxygen species in cisplatin-resistant CAR human oral cancer cells. Int J Oncol (2013) 43:1141–50. doi: 10.3892/ijo.2013.2050 23917396

[31] Abdallah B Hassan A Benoist C Goula D Behr JP Demeneix BA. A powerful nonviral vector for in *vivo* gene transfer into the adult mamMalian brain: polyethylenimine. Hum Gene Ther (1996) 7:1947–54. doi: 10.1089/hum.1996.7.16-1947 8930654

[32] Rhaese S Von Briesen H Rübsamen-Waigmann H Kreuter J Langer K. Human serum albumin–polyethylenimine nanoparticles for gene delivery. J Controlled Release (2003) 92:199–208. doi: 10.1016/S0168-3659(03)00302-X 14499197

[33] Urban-Klein B Werth S Abuharbeid S Czubayko F Aigner A. RNAi-mediated gene-targeting through systemic application of polyethylenimine (PEI)-complexed siRNA in *vivo* . Gene Ther (2005) 12:461–6. doi: 10.1038/sj.gt.3302425 15616603

[34] Meng H Liong M Xia T Li Z Ji Z Zink JI. Engineered design of mesoporous silica nanoparticles to deliver doxorubicin and P-glycoprotein siRNA to overcome drug resistance in a cancer cell line. ACS nano (2010) 4:4539–50. doi: 10.1021/nn100690m PMC3899722 20731437

[35] Navarro G Sawant RR Biswas S Essex S Tros de Ilarduya C Torchilin VP. P-glycoprotein silencing with siRNA delivered by DOPE-modified PEI overcomes doxorubicin resistance in breast cancer cells. Nanomedicine (2012) 7:65–78. doi: 10.2217/nnm.11.93 22191778 PMC3422569

[36] Yang Y-P Chien Y Chiou G-Y Cherng J-Y Wang M-L Lo W-L. Inhibition of cancer stem cell-like properties and reduced chemoradioresistance of glioblastoma using microRNA145 with cationic polyurethane-short branch PEI. Biomaterials (2012) 33:1462–76. doi: 10.1016/j.biomaterials.2011.10.071 22098779

[37] Ghodbane S Lahbib A Sakly M Abdelmelek H. Bioeffects of static magnetic fields: oxidative stress, genotoxic effects, and cancer studies. BioMed Res Int (2013) 2013:602987. doi: 10.1155/2013/602987 24027759 PMC3763575

[38] Hajipour B Alipour M Abdolmaleki P Behmanesh M. Magnetic field exposure alters the expression of DNA repair genes. J Cell Immunother (2017) 3:3. doi: 10.1016/j.jocit.2017.04.004

[39] Jalali A Zafari J Jouni FJ Abdolmaleki P Shirazi FH Khodayar MJ. Combination of static magnetic field and cisplatin in order to reduce drug resistance in cancer cell lines. Int J Radiat Biol (2019) 95:1194–201. doi: 10.1080/09553002.2019.1589012 30822212

[40] Kamalipooya S Soleimani H Abdolmaleki P Sabet A Hajipour B Jouni FJ. The effects of static magnetic fields on viability and apoptosis in normal and cancerous cells. Stress (2015) 1:86–90.

[41] El Ghandoor H Zidan H Khalil MM Ismail M. Synthesis and some physical properties of magnetite (Fe3O4) nanoparticles. Int J Electrochem Sci (2012) 7:5734–45. doi: 10.1016/S1452-3981(23)19655-6

[42] Kazemi-Ashtiyani M Hajipour-Verdom B Satari M Abdolmaleki P Hosseinkhani S Shaki H. Estimating the two graph dextran–stearic acid–spermine polymers based on iron oxide nanoparticles as carrier for gene delivery. Biopolymers (2022) 113:e23491. doi: 10.1002/bip.23491 35560028

[43] Jawad AS Al-Alawy AF. Synthesis and characterization of coated magnetic nanoparticles and its application as coagulant for removal of oil droplets from oilfield produced water. AIP Conf Proc (2020) 2213:020174. doi: 10.1063/5.0000279

[44] Ullah R Faisal M Ullah R. Molecular and biomolecular spectroscopy. Spectrochimica Acta Part A: Mol Biomolecular Spectrosc (2023) 293:122490. doi: 10.1016/j.saa.2023.122490 36801738

[45] An J Shi K Wei W Hua F Ci Y Jiang Q. The ROS/JNK/ATF2 pathway mediates selenite-induced leukemia NB4 cell cycle arrest and apoptosis in *vitro* and in *vivo* . Cell Death Dis (2013) 4:e973–3. doi: 10.1038/cddis.2013.475 PMC3877548 24357804

[46] Riccardi C Nicoletti I. Analysis of apoptosis by propidium iodide staining and flow cytometry. Nat Protoc (2006) 1:1458–61. doi: 10.1038/nprot.2006.238 17406435

[47] Alipour M Hajipour-Verdom B Javan M Abdolmaleki P. Static and electromagnetic fields differently affect proliferation and cell death through acid enhancement of ROS generation in mesenchymal stem cells. Radiat Res (2022) 198:384–95. doi: 10.1667/RADE-21-00037.1 35867630

[48] Shapiro HM. Practical flow cytometry. New York, NY, USA: John Wiley & Sons (2005).

[49] Tseng C-L Yang K-C Yen K-C Yueh-Hsiu Wu S Lin F-H. Preparation and characterization of cisplatin-incorporated gelatin nanocomplex for cancer treatment. Curr Nanoscience (2011) 7:932–7. doi: 10.2174/157341311798220736

[50] Mainini F Eccles MR. Lipid and polymer-based nanoparticle siRNA delivery systems for cancer therapy. Molecules (2020) 25:2692. doi: 10.3390/molecules25112692 32532030 PMC7321291

[51] Yang T Du G Cui Y Yu R Hua C Tian W. pH-sensitive doxorubicin-loaded polymeric nanocomplex based on β-cyclodextrin for liver cancer-targeted therapy. Int J Nanomed (2019) 14:1997–2010. doi: 10.2147/IJN.S193170 PMC6433111 30962684

[52] Lim WQ Phua SZF Zhao Y. Redox-responsive polymeric nanocomplex for delivery of cytotoxic protein and chemotherapeutics. ACS Appl materials interfaces (2019) 11:31638–48. doi: 10.1021/acsami.9b09605 31389684

[53] Marullo R Werner E Degtyareva N Moore B Altavilla G RaMalingam SS. Cisplatin induces a mitochondrial-ROS response that contributes to cytotoxicity depending on mitochondrial redox status and bioenergetic functions. PloS One (2013) 8:e81162. doi: 10.1371/journal.pone.0081162 24260552 PMC3834214

[54] Chen HH Kuo MT. Role of glutathione in the regulation of Cisplatin resistance in cancer chemotherapy. Metal-based Drugs (2010) 2010:1–7. doi: 10.1155/2010/430939 PMC2946579 20885916

[55] Khansarizadeh M Mokhtarzadeh A Rashedinia M Taghdisi S Lari P Abnous K. Identification of possible cytotoxicity mechanism of polyethylenimine by proteomics analysis. Hum Exp Toxicol (2016) 35:377–87. doi: 10.1177/0960327115591371 26134983

[56] Karimi F Fallah Shojaei A Tabatabaeian K Karimi-Maleh H Shakeri S. HSA loaded with CoFe2 O4/MNPs as a high-efficiency carrier for epirubicin anticancer drug delivery. IET Nanobiotechnol (2018) 12:336–42. doi: 10.1049/iet-nbt.2017.0057

[57] Basu A Krishnamurthy S. Cellular responses to Cisplatin-induced DNA damage. J Nucleic Acids (2010) 2010:201367. doi: 10.4061/2010/201367 20811617 PMC2929606

[58] Valko M Rhodes C Moncol J Izakovic M Mazur M. Free radicals, metals and antioxidants in oxidative stress-induced cancer. Chemico-biological Interact (2006) 160:1–40. doi: 10.1016/j.cbi.2005.12.009 16430879

[59] Goodarzi AA Noon AT Deckbar D Ziv Y Shiloh Y Löbrich M. ATM signaling facilitates repair of DNA double-strand breaks associated with heterochromatin. Mol Cell (2008) 31:167–77. doi: 10.1016/j.molcel.2008.05.017 18657500

[60] Zhang Y Qian D Li Z Huang Y Wu Q Ru G. Oxidative stress-induced DNA damage of mouse zygotes triggers G2/M checkpoint and phosphorylates Cdc25 and Cdc2. Cell Stress Chaperones (2016) 21:687–96. doi: 10.1007/s12192-016-0693-5 PMC4907999 27117522

[61] Cao X Hou J An Q Assaraf YG Wang X. Towards the overcoming of anticancer drug resistance mediated by p53 mutations. Drug Resistance Updates (2020) 49:100671. doi: 10.1016/j.drup.2019.100671 31841768

[62] Chen L Chen C Wang P Song T. Mechanisms of cellular effects directly induced by magnetic nanoparticles under magnetic fields. J nanomaterials (2017) 2017:1–13. doi: 10.1155/2017/1564634

[63] Latunde-Dada GO. Ferroptosis: role of lipid peroxidation, iron and ferritinophagy. Biochim Biophys Acta (BBA)-General Subj (2017) 1861:1893–900. doi: 10.1016/j.bbagen.2017.05.019 28552631

[64] Santana-Codina N Mancias JD. The role of NCOA4-mediated ferritinophagy in health and disease. Pharmaceuticals (2018) 11:114. doi: 10.3390/ph11040114 30360520 PMC6316710

[65] Xie J Yang Y Gao Y He J. Cuproptosis: mechanisms and links with cancers. Mol Cancer (2023) 22:46. doi: 10.1186/s12943-023-01732-y 36882769 PMC9990368

[66] Peng Q Jiang X Tan S Xu X Xia L Wu N. Clinical significance and integrative analysis of the cuproptosis-associated genes in head and neck squamous cell carcinoma. Aging (Albany NY) (2023) 15:1964. doi: 10.18632/aging.204579 36947706 PMC10085596

[67] Zhu Y Sui B Liu X Sun J. The reversal of drug resistance by two-dimensional titanium carbide Ti2C (2D Ti2C) in non-small-cell lung cancer via the depletion of intracellular antioxidant reserves. Thorac Cancer (2021) 12:3340–55. doi: 10.1111/1759-7714.14208 PMC8671908 34741403

[68] Bernareggi A Torti L Facino RM Carini M Depta G Casetta B. Characterization of cisplatin-glutathione adducts by liquid chromatography-mass spectrometry evidence for their formation in *vitro* but not in *vivo* after concomitant administration of cisplatin and glutathione to rats and cancer patients. J Chromatogr B: Biomed Sci Appl (1995) 669:247–63. doi: 10.1016/0378-4347(95)00098-4 7581901

[69] Peklak-Scott C Smitherman PK Townsend AJ Morrow CS. Role of glutathione S-transferase P1-1 in the cellular detoxification of cisplatin. Mol Cancer Ther (2008) 7:3247–55. doi: 10.1158/1535-7163.MCT-08-0250 PMC2587032 18852128

[70] Bodega G Alique M Puebla L Carracedo J Ramírez R. Microvesicles: ROS scavengers and ROS producers. J extracellular vesicles (2019) 8:1626654. doi: 10.1080/20013078.2019.1626654 31258880 PMC6586107

[71] Boussif O Lezoualc'h F Zanta MA Mergny MD Scherman D Demeneix B. A versatile vector for gene and oligonucleotide transfer into cells in culture and in *vivo*: polyethylenimine. Proc Natl Acad Sci (1995) 92:7297–301. doi: 10.1073/pnas.92.16.7297 PMC41326 7638184

[72] Benjaminsen RV Mattebjerg MA Henriksen JR Moghimi SM Andresen TL. The possible “proton sponge” effect of polyethylenimine (PEI) does not include change in lysosomal pH. Mol Ther (2013) 21:149–57. doi: 10.1038/mt.2012.185 PMC3538306 23032976

