# Supplementary material for: Polyethylenimine-based iron oxide nanoparticles enhance cisplatin toxicity in ovarian cancer cells in the presence of a static magnetic field
Source: Front Oncol. 2023 Sep 12;13:1217800. doi: 10.3389/fonc.2023.1217800 (PMC10522916; doi:10.3389/fonc.2023.1217800)
Supplement: Supplementary file 1 [file DataSheet_1.pdf]

Supplementary Information for:

Polyethylenimine-based magnetic nanocomplexes enhanced cisplatin toxicity on ovarian cancer cells in presence of static magnetic field

Faranak ashoori[1], Behnam Hajipour-Verdom[1], Mohammad Satari[2], Parviz Abdolmaleki[1,*].

[1] *Department of Biophysics, Faculty of Biological Sciences, Tarbiat Modares University, Tehran, 14115-154, Iran.*

[2] *Department of Biology, Faculty of Basic Sciences, Malayer University, Malayer, 65719-95863, Iran.*

***Corresponding Author:** Parviz Abdolmaleki, Professor of Biophysics, Faculty of Biological Sciences, Tarbiat Modares University, Tehran, 14115-154, Iran, Tel.: +98 21 8288 3404. E-mail: parviz@modares.ac.ir

Supplementary Information

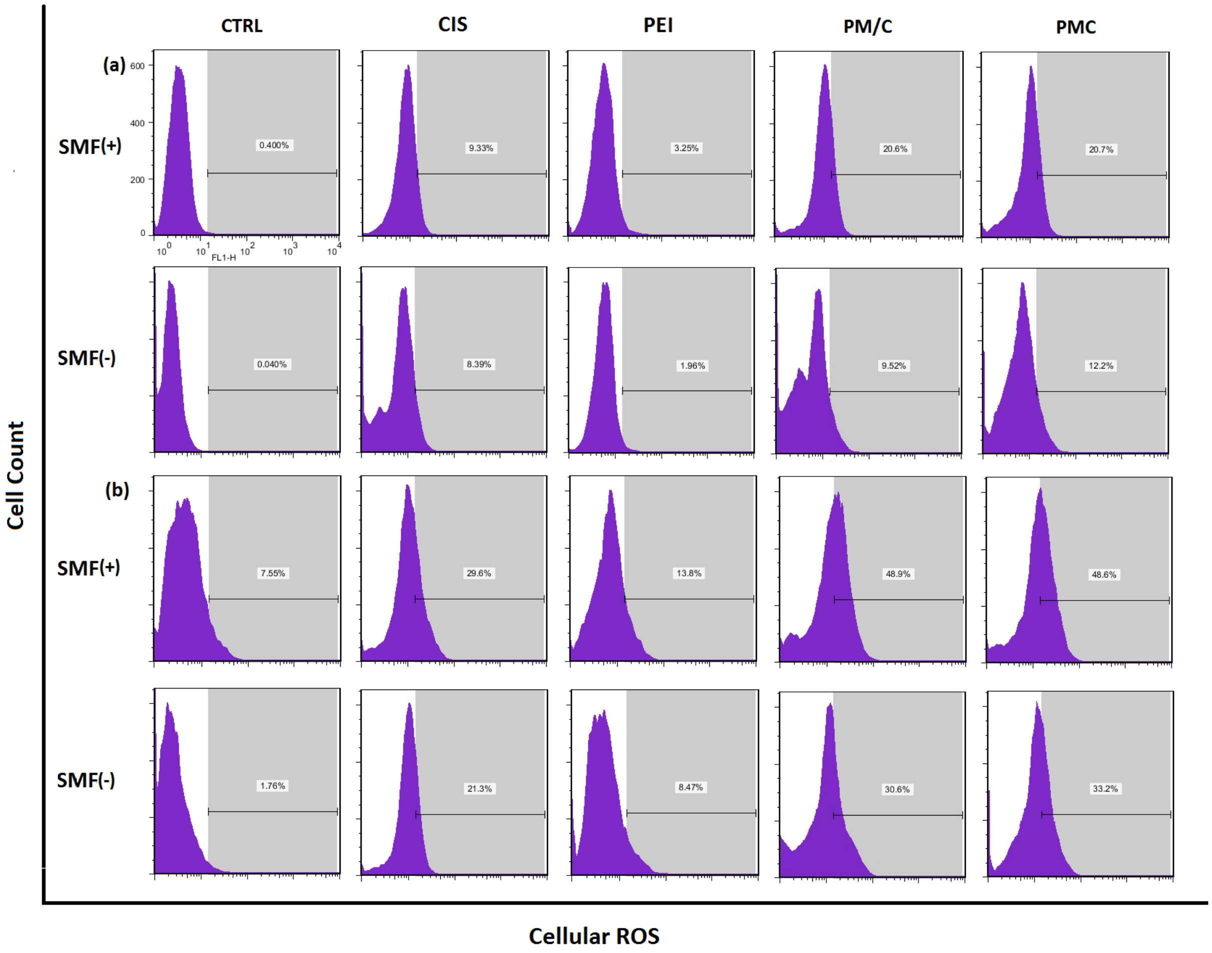

Supplementary Figure S1. The flow cytometry analysis for intracellular ROS generation of **(a)** A2780/CP and **(b)** A2780 cells treated to 1 µg/ml of polyethylenimine (PEI), 2.5 µg/ml of cisplatin (CIS), and PM/C and PMC three-component magnetic nanocomplexes at same concentrations (1 µg/ml PEI, 1 µg/ml MNPs and 2.5 µg/ml CIS) in presence and absence of 20 mT static magnetic field (SMF) for 48 h. Cells were collected and evaluated by oxidized DCFDA assay kit.

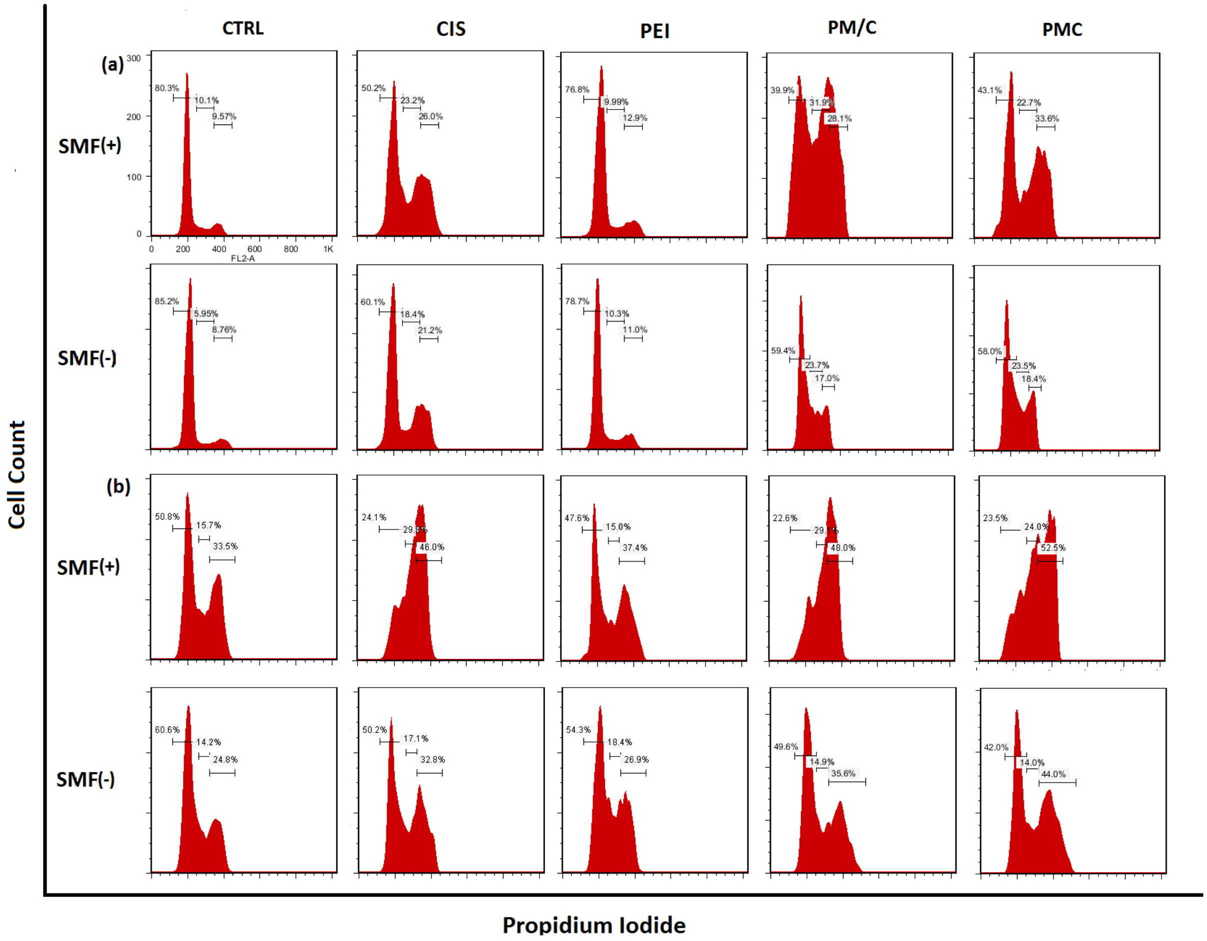

Supplementary Figure S2. The flow cytometry analysis for cell cycle distribution of **(a)** A2780/CP and **(b)** A2780 cells treated to 1 µg/ml of polyethylenimine (PEI), 2.5 µg/ml of cisplatin (CIS), and PM/C and PMC three-component magnetic nanocomplexes at same concentrations (1 µg/ml PEI, 1 µg/ml MNPs and 2.5 µg/ml CIS) in presence and absence of 20 mT static magnetic field (SMF) for 48 h. Cells were collected and analyzed by propidium iodide flow cytometry kit.

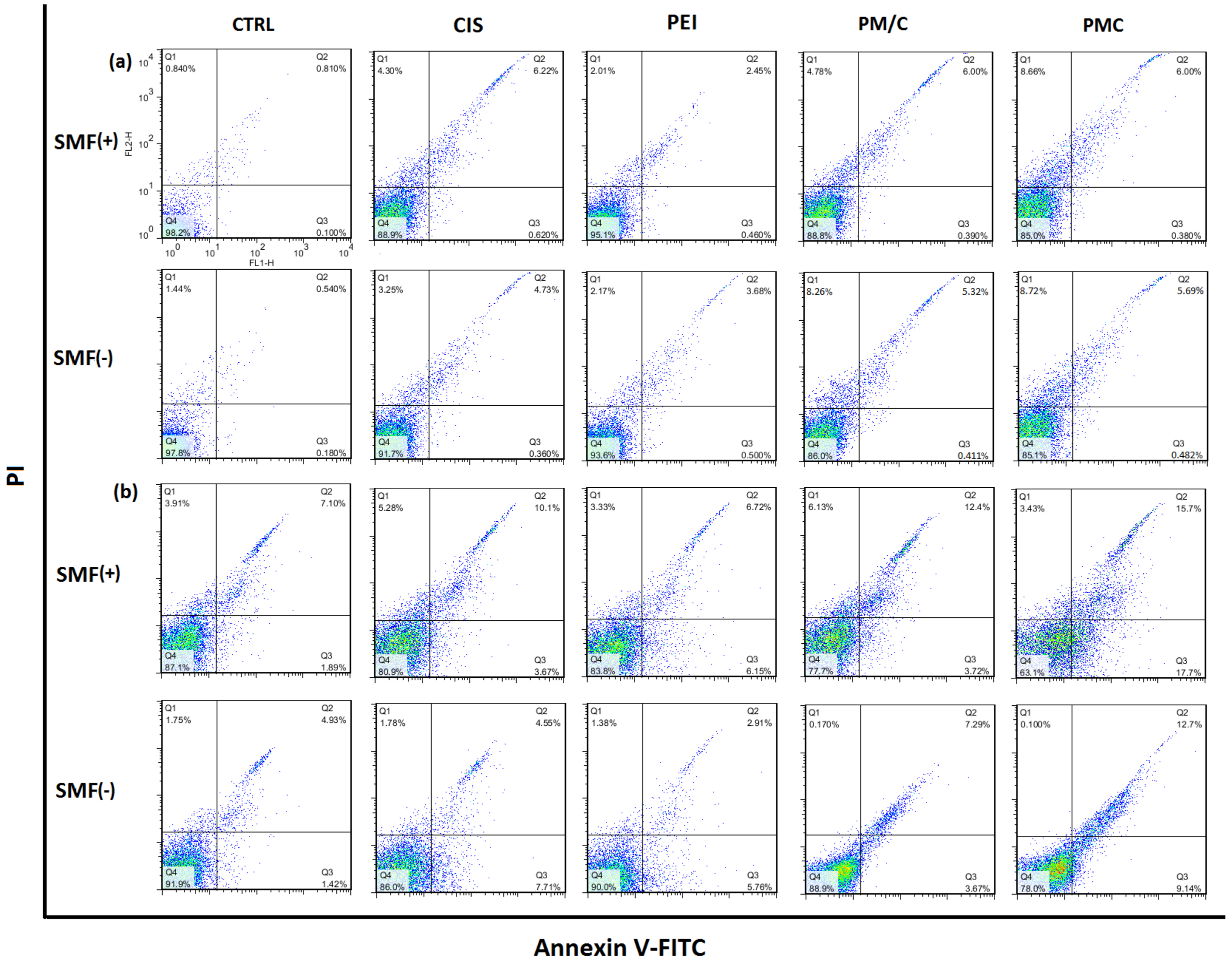

Supplementary Figure S3. The flow cytometry analysis for apoptosis cell death of **(a)** A2780/CP and **(b)** A2780 cells treated to 1 µg/ml of polyethylenimine (PEI), 2.5 µg/ml of cisplatin (CIS), and PM/C and PMC three-component magnetic nanocomplexes at same concentrations (1 µg/ml PEI, 1 µg/ml MNPs and 2.5 µg/ml CIS) in presence and absence of 20 mT static magnetic field (SMF) for 48 h. Cells were collected and labeled with annexin-V/PI kit.
